# Epidemiological characteristics and genetic diversity of norovirus infections among outpatient children with diarrhea under 5 years of age in Beijing, China, 2011–2018

**DOI:** 10.1186/s13099-021-00473-x

**Published:** 2021-12-24

**Authors:** Weihong Li, Hanqiu Yan, Baiwei Liu, Yi Tian, Yanwei Chen, Lei Jia, Zhiyong Gao, Quanyi Wang

**Affiliations:** Institute for Infectious Disease and Endemic Disease Control, Beijing Center for Disease Prevention and Control and Beijing Research Center for Preventive Medicine, Beijing, China

**Keywords:** Norovirus, Children, Outpatient, Diarrhea, Genotypes

## Abstract

**Background:**

Human noroviruses are the leading cause of sporadic cases and outbreaks of viral acute gastroenteritis in all age groups worldwide.

**Methods:**

Epidemiological data and fecal specimens were collected between January 2011 and December 2018 from 4911 children < 5 years of age with diarrhea in three districts of Beijing. From 2011 to 2013, One-Step Reverse Transcription Polymerase Chain Reaction (RT-PCR) was used to detect noroviruses, and from January 2014 to December 2018, norovirus GI and GII were screened using duplex quantitative real-time RT-PCR (qRT-PCR). One-Step RT-PCR and RT-seminested PCR were performed to amplify the RNA-dependent polymerase and capsid genes of noroviruses in positive sample. Amplified products were sequenced directly; norovirus was typed using the online Norovirus Genotyping Tool v2.0 and phylogenetic analyses were conducted using MEGA-X.

**Results:**

From 2011 to 2018, noroviruses were detected in 16.5% of specimens from children with diarrhea. The highest prevalence was observed in children aged 12 to 23 months (22.4%, 319/1421), followed by children aged 6 to 11 months (17.6%, 253/1441). The highest prevalence of norovirus infections occurred in autumn followed by winter, spring, and summer. From 2011 to 2018, the most prevalent dual types (genotype and polymerase type) were GII.4 Sydney[P31] (51.6%, 239/463), followed by GII.3[P12] (24.0%, 111/463), GII.4 2006b[P4 2006b] (7.3%, 34/463), GII.2[P16] (5.0%, 23/463), GII.17[P17] (2.6%, 12/463) and GII.6[P7] (2.6%, 12/463). GII.4 2006b[P4 2006b] predominated in 2011 and 2012. GII.4 Sydney[P31] predominated from 2013 to 2018. In total, 15 genotypes, 15 P-types and 19 dual types were detected in this study, reflecting the genetic diversity.

**Conclusions:**

There were significant epidemiological characteristics and genetic diversity among outpatient children with norovirus infections < 5 years of age in Beijing from 2011 to 2018. These characteristics differ from those of norovirus outbreaks in Beijing. The complete genome sequences of each genotype are needed to better understand norovirus evolutionary mechanisms.

## Background

Despite substantially decreased incidence in recent decades, diarrheal disease remains the second most common cause of morbidity in children <5 years of age and is associated with 500,000 deaths each year worldwide [1, 2]. Noroviruses are the leading cause of acute gastroenteritis (AGE) in all age groups worldwide [3, 4].

With the introduction of rotavirus vaccines, noroviruses are now the most common cause of pediatric AGE in some countries [5, 6]. A nationwide etiological study of AGE in China conducted between 2009 to 2013 (a time when rotavirus vaccines were not included in the national immunization program) [7] found that norovirus was the second most common enteropathogen among children <5 years of age [8]. However, because AGE caused by noroviruses is self-limiting, and children with AGE experiencing vomiting only were excluded from nearly all studies, published data almost certainly underestimate the burden of norovirus infection in China [9].

Noroviruses belong to the *Norovirus* genus in the *Caliciviridae* family and are non-enveloped single-stranded positive-sense RNA viruses. The norovirus genome is about 7.5 kb in size and encodes three open reading frames (ORFs). ORF1 encodes a large polyprotein that is posttranslationally cleaved into at least six nonstructural proteins including the RNA-dependent RNA polymerase (RdRp). ORF2 encodes the major capsid protein (VP1) and ORF3 encodes a minor capsid protein (VP2) [10].

Noroviruses were officially subdivided into six genogroups (GI–GVI) in 2013 [11]. A tentative genogroup GVII was proposed in 2015 based on analysis of amino acid sequence diversity in the complete VP1 capsid protein [12]. In 2019, the number of norovirus genogroups was expanded to 10 (GI–GX) and the number of genotypes was expanded to 48 based on the complete capsid amino acid sequences. Moreover, 60 P-types were identified based on the partial nucleotide sequences of RdRp regions [13]. Norovirus classification was recently updated to be based on dual typing (genotype and polymerase type) [4, 13]. GI, GII, GIV, GVIII, and GIX noroviruses are associated with human infections [13]. GII noroviruses are responsible for most sporadic cases and outbreaks of AGE, followed by GI noroviruses. GIV, GVIII and GIX noroviruses rarely cause AGE [13–15].

In sporadic cases of children < 5 years of age, norovirus genotypes differ from those responsible for outbreaks [16–19]. In a Japanese survey during 2002–2011 [16], Sakon and colleagues reported that in sporadic pediatric cases, the GII.4 genotype was predominant in 8 of the 10 seasons and GII.3 dominated 2 seasons; however, the dominant genotypes in outbreaks at childcare facilities and schools shifted every season and involved GII.1, GII.2, GII.3, GII.4, and GII.6. In the past 10 years, norovirus outbreaks caused by GII.4 Sydney strains, GII.17[P17] 2014–2015 strains and GII.2[P16] 2016–2017 strains have been reported in several provinces of China [20–26], and these outbreaks mainly occurred in kindergartens and primary schools. The characteristics of noroviruses responsible for sporadic pediatric cases over the same years have not been systematically reported [27–29] except that in Shanghai [19], impeding understanding of differences in genotype diversity between noroviruses causing sporadic cases and outbreaks. In Beijing, norovirus genotypes among outpatients (mainly adults) with diarrhea between 2011 and 2013 and outbreaks from 2014 to 2017 have been reported [23, 26, 28]. However, corresponding epidemiological characteristics and genetic diversity of norovirus in sporadic cases of children < 5 years of age from 2011 to 2018 are lacking.

Since 2011, a hospital-based surveillance network for sporadic diarrhea in children < 5 years of age has been operating in three districts of Beijing. In this study, we analyzed the epidemiologic and genetic features of noroviruses among outpatient children < 5 years of age from 2011 to 2018 using this network. The genotype diversity of norovirus in sporadic cases of children < 5 years of age was compared with those in outbreaks during 2014–2017 in Beijing [23] and 2016–2018 in China [18]. These data may further improve our understanding of norovirus infection.

## Methods

### Case definition

Cases were defined as outpatients < 5 years of age with diarrhea (three or more loose stools within a 24-h period).

### Surveillance and sampling

Starting in 2011, four children’s hospitals located in three districts (Xicheng, Tongzhou, and Chaoyang) were chosen as sentinel hospitals in the surveillance network for sporadic diarrhea in children < 5 years old in Beijing. Stool specimens and sociodemographic data were collected from children < 5 years of age newly diagnosed with acute diarrhea. Approximately 15 specimens were collected in each district every month. District-level Centers for Disease Control and Prevention (CDCs) were responsible for collecting specimens from sentinel hospitals since January 2011 and they conducted norovirus screening using conventional reverse transcription PCR (RT-PCR) since January 2011 and genotype-specific real-time RT-PCR (qRT-PCR) since January 2014. Norovirus-positive specimens were sent to the Beijing CDC, sequencing and genotyping were performed.

### RNA extraction

10% (w/v) fecal suspensions were prepared with phosphate-buffered saline. The suspensions were thoroughly vortexed and then centrifuged at 8000 × g for 5 min. Viral RNA was extracted from 140 µL of the supernatant using the QIAamp Viral RNA Mini Kit (Qiagen, Hilden, Germany) according to the manufacturer’s protocol. RNA was stored at -20 °C until use.

### Norovirus detection and genotyping

From January 2011 to December 2013, One-Step reverse transcription polymerase chain reaction (RT-PCR) was used to detected norovirus. Using the primer pair 290/289 targeting the RdRp gene, a 319-bp PCR product was generated [30]. Primers G1SKF/G1SKR and COG2F/G2SKR were used to amplify the partial VP1 genes of norovirus GI and norovirus GII [31–33]. RT-seminested PCR was carried out for genotyping using primer pairs COG1F/G1SKR and G1SKF/G1SKR for norovirus GI, COG2F/G2SKR and G2SKF/G2SKR for norovirus GII, generating 330-bp and 344-bp PCR products[31–33].

From January 2014 to December 2018, the presence of noroviruses was detected using a genotype-specific qRT-PCR kit for the detection of norovirus GI and GII (Bioperfectus technologies Co., Ltd, Jiangsu, China). This kit targets the ORF1-ORF2 junction of noroviruses. From January 2014 to August 2016, positive samples were further analyzed by amplification of partial RdRp and VP1 regions using the QIAGEN OneStep RT-PCR Kit with the same primers as mentioned above. Starting in September 2016, positive samples were further analyzed by amplification of the ORF1-ORF2 junction of the norovirus genome using the QIAGEN OneStep RT-PCR Kit with primer pairs MON432/G1SKR and MON431/G2SKR[34, 35]. RT-PCR was performed using the following thermal cycling parameters: 50 °C for 30 min; 95 °C for 15 min; 40 cycles of 94 °C for 30 s, 54 °C for 1 min, and 72 °C for 1 min; and a final extension at 72 °C for 7 min. The resulting amplicons were 579 bp for norovirus GI and 570 bp for norovirus GII. RT-seminested PCR using MON432/G1SKR and G1SKF/G1SKR for norovirus GI, MON431/G2SKR and COG2F/G2SKR for norovirus GII were used for genotyping if necessary.

All PCR products were analyzed using the QIAxcel DNA Screening Kit (Qiagen). PCR products from norovirus positive fecal specimens were subjected to nucleotide sequencing (Sangon Biotech Co., Ltd., Shanghai, China) and then genotyped using the RIVM online norovirus genotyping tool (https://www.rivm.nl/mpf/norovirus/typingtool). Nucleotide sequences were deposited in GenBank under accession numbers MW686549–MW6728, MW686747, and MW686748.

### Phylogenetic analysis

From 2011 to 2018, 282 bp partial capsid gene sequences were obtained using primer pairs G1SKF/G1SKR and Mon432 /G1SKR for GI noroviruses and primer pairs G2SKF/G2SKR and Mon431/G2SKR for GII noroviruses. The 274 bp partial RdRp sequences obtained using the primer pair 289/290 between 2011 and 2016 did not overlap those obtained using primer pairs Mon432/G1SKR and Mon431/G2SKR during 2016–2018. Thus, three phylogenetic trees were constructed based on the 282 bp capsid gene sequences from 2011 to 2018, the 274 bp partial RdRp sequences from 2011 to 2015, and the 262 bp partial RdRp sequences from 2016 to 2018, respectively. Phylogenetic trees were constructed using the maximum likelihood method implemented in MEGA-X software with 1000 bootstrap replicates. The best nucleotide substitution model producing the lowest Bayesian information criterion was determined using the maximum likelihood model testing tool. The K2 + G (Kimura two-parameter method and gamma distribution) model was selected as the best fit model.

### Data analysis

Epidemiological and detection data for all diarrhea cases were collected and subjected to statistical analyses with SPSS v21.0 software (SPSS Inc., Chicago, IL, USA). According to the date of onset, cases were divided into four groups: spring (March–May), summer (June–August), autumn (September–November), and winter (December–February). The chi-square test with a two-sided significance level of 0.05 was used to assess the differences of positive rates between genders, age groups, places of residence, years, and seasons. The chi-square test with a two-sided significance level of 0.0083 (with Bonferroni adjustment for multiple comparisons) was further used for the pairwise comparisons of positive rates between each two different seasons.

### Ethics statement

The study was approved by the Ethics Committee of the Beijing CDC.

## Results

### Noroviruses in children with diarrhea under 5 years of age

From January 2011 to December 2018, a total of 4911 stool specimens were collected from outpatient children with diarrhea < 5 years of age (Table 1). Among these outpatients, 3063 (62.4%) were boys, 1848 (37.6%) were girls, and 809 (16.5%) were infected with noroviruses. No significant difference in norovirus prevalence was observed between boys (16.8%, 516/3063) and girls (15.9%, 293/1848).

**Table 1:** Demographic characteristics of norovirus infections among outpatient children with diarrhea in Beijing, 2011–2018

Demographic characteristics	Total number	Number of Norovirus positive (%)	OR	95%*CI*	*P*-value
Gender					
Male	3063	516 (16.8%)	1.00	Reference	
Female	1848	293 (15.9%)	0.93	0.80–1.09	0.364
Age (months)					
0–5	1292	130 (10.1%)	1.00	Reference	
6–11	1441	253 (17.6%)	1.90	1.51–2.39	< 0.01
12–23	1421	319 (22.4%)	2.59	2.08–3.22	< 0.01
24–35	400	59 (14.8%)	1.55	1.11–2.15	0.009
36–47	225	37 (16.4%)	1.76	1.18–2.62	0.005
48–59	132	11 (8.3%)	0.81	0.43–1.55	0.527
Living area					
Urban	3323	542 (16.3%)	1.00	Reference	
Rural	1588	267 (16.8%)	1.04	0.88–1.22	0.657
Year					
2011	596	113 (19.0%)	1.00	Reference	
2012	652	112 (17.2%)	0.89	0.66–1.18	0.419
2013	639	95 (14.9%)	0.75	0.55–1.01	0.057
2014	548	85 (15.5%)	0.78	0.58–1.07	0.137
2015	570	89 (15.6%)	0.79	0.58–1.07	0.142
2016	718	107 (14.9%)	0.75	0.56–1.00	0.054
2017	621	107 (17.2%)	0.89	0.66–1.19	0.457
2018	567	101 (17.8%)	0.93	0.69–1.25	0.650
Season					
Spring	1225	180 (14.7%)	0.75	0.60–0.93	0.009
Summer	1280	121 (9.5%)	0.45	0.36–0.58	< 0.001
Autumn	1250	292 (23.3%)	1.33	1.09–1.62	0.005
Winter	1156	216 (18.7%)	1.00	Reference	

The overall prevalence of norovirus in different age groups ranged from 8.3% to 22.4%. Children aged 12 to 23 months had the highest prevalence (22.4%, 319/1421) followed by children aged 6 to 11 months (17.6%, 253/1441). Most children infected with noroviruses were younger than 3 years of age (94.1%, 761/809). Norovirus prevalence was similar among children living in urban and rural areas.

**Fig. 1:**
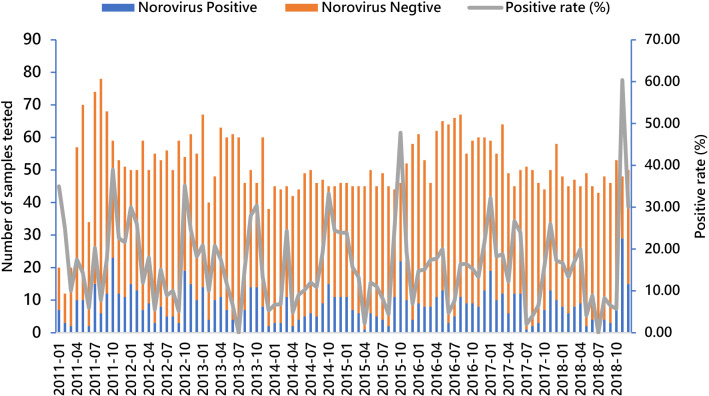
Monthly distribution of norovirus infections among outpatient children with diarrhea in Beijing, 2011–2018

The monthly prevalence of norovirus ranged from 0.0% to 60.4% between 2011 and 2018 (Fig. 1). From 2011 to 2015, the prevalence peak occurred in October. In 2016 and 2017, peak prevalence occurred in December and January, respectively. In 2018, peak prevalence occurred in November. The season with the highest prevalence of norovirus infection was autumn (23.3%, 292/1250) followed by winter, spring, and summer (Table 1). Significant difference of positive rates was found between seasons (χ^2^ = 95.86, *P* < 0.001). Further pairwise comparisons of positive rates between each two different seasons revealed that there were significant differences of positive rates between each two different seasons except that between spring and winter (the significant level of 0.0083 with Bonferromi adjustment was used).

The annual prevalence of norovirus ranged from 14.9% to 19.0% between 2011 and 2018. No significant differences were found between years (χ^2^ = 7.072, *P* > 0.05).

From January 2011 to December 2013, a total of 1887 stool specimens were collected, among which 320 (17.0%) specimens were positive for noroviruses by RT-PCR. A total of 57.5% (184/320) of the number of positive samples were successfully sequenced. All were identified as GII noroviruses.

From January 2014 to December 2018, qRT-PCR screening was carried out. During this period, a total of 3024 stool specimens were collected and 489 (16.2%) specimens were positive for noroviruses. Among these noroviruses, 475 (97.1%) were identified as GII, 10 (2.0%) were identified as GI, and 4 (0.8%) were identified as GI/GII mixed infection. The overall prevalence of GI, GII, and GI/GII norovirus mixed infection was 0.3% (10/3024), 15.7% (475/3024) and 0.1% (4/3024), respectively.

### Norovirus genotype analysis based on partial capsid region sequences

From 2011 to 2018, we detected 15 genotypes in 66.7% (540/809) of positive cases. The most common genotypes were GII.4 Sydney (46.1%, 249/540) followed by GII.3 (26.5%, 143/540), GII.2 (8.0%, 43/540), GII.4 2006b (7.2%, 39/540), GII.17 (3.5%, 19/540), GII.6 (3.3%, 18/540), GII.4 unassigned (2.0%, 11/540), and 8 other genotypes each accounting for less than 1.0% of all noroviruses. From 2013 to 2018, GII.4 Sydney was the most prevalent genotype. GII.4 2006b was the most prevalent genotype in 2011 and 2012.

### Norovirus P-type analysis based on partial RdRp region sequences

From 2011 to 2018, we detected 15 P-types in 61.1% (494/809) of positive cases. The most common P-types were GII.P31 (50.0%, 245/494) followed by GII.P12 (24.1%, 119/494), GII.P4 2006b (9.7%, 48/494), GII.P16 (5.1%, 25/494), GII.P7 (4.5%, 22/494), GII.P17 (2.8%, 14/494), GII.P2 (1.2%, 6/494), and 8 other P-types accounting for less than 1.0% of all noroviruses. GII.P4 2006b was the most common P-type in 2011 and 2012. From 2013 to 2018, GII.P31 was the predominant P-type.

### Norovirus dual type analysis based on RdRp/capsid gene sequences

As shown in Table 2, we detected 19 dual types in 57.2% (463/809) of positive cases from 2011 to 2018. The five most prevalent dual types were GII.4 Sydney[P31] (51.6%, 239/463) followed by GII.3[P12] (24.0%, 111/463), GII.4 2006b[P4 2006b] (7.3%, 34/463), GII.2[P16] (5.0%, 23/463),and GII.17[P17] (2.6%, 12/463). In 2011 and 2012, the predominant dual type was GII.4 2006b[P4 2006b] (50.0%, 14/28 in 2011; 34.5%, 20/58 in 2012). From 2013 to 2018, GII.4 Sydney[P31] was the predominant dual type, making up 49.1% to 76.2% of all typed strains in each year. The second most prevalent dual type from 2013 to 2018 was GII.3[P12] (9.5%–45.6%), except in 2015 when GII.17[P17] was the same prevalent as GII.3[P12] (9.5%, 6/63) and in 2017 when GII.2[P16] was more prevalent (27.7%, 20/72).

**Table 2:** Norovirus dual types among outpatient children with diarrhea in Beijing, 2011–2018

Genotype[P-type]	2011 N(m%)	2012 N(m%)	2013 N(m%)	2014 N(m%)	2015 N(m%)	2016 N(m%)	2017 N(m%)	2018 N(m%)	Total N(m%)
GI.5[P4]	–	–	–	–	–	–	–	1(1.7)	1(0.2)
GII.1[P16]	–	–	–	–	–	–	–	1(1.7)	1(0.2)
GII.1[P33]	–	–	1(1.8)	–	–	–	–	–	1(0.2)
GII.2[P2]	–	–	-	3(4.9)	1(1.6)	2(3.1)	–	–	6(1.3)
GII.2[P12]	–	–	-	1(1.6)	–	–	–	–	1(0.2)
GII.2[P16]	–	–	-	–	–	–	20(27.7)	3(5.1)	23(5.0)
GII.3[P12]	6(21.4)	18(31.0)	26(45.6)	13(21.3)	6(9.5)	17(26.2)	12(16.7)	13(22.0)	111(24.0)
GII.3[P21]	2(7.1)	-	–	–	–	–	–	–	2(0.4)
GII.3[P4 2006b]	2(7.1)	-	–	–	–	–	–	–	2(0.4)
GII.4 2006b[P4 2006b]	14(50.0)	20(34.5)	–	–	–	–	–	–	34(7.3)
GII.4 unassigned[P4 2006b]	3(10.7)	5(8.6)	–	–	–	–	–	–	8(1.7)
GII.4 2009[P4 2009]	1(3.6)	2(3.4)	–	–	–	–	–	–	3(0.6)
GII.4 Sydney[P31]	–	13(22.4)	28(49.1)	38(62.3)	48(76.2)	36(55.4)	39(54.2)	37(62.7)	239(51.6)
GII.6[P7]	–	-	2(3.5)	5(8.2)	1(1.6)	3(4.6)	–	1(1.7)	12(2.6)
GII.7[P7]		–	–	–	–	4(6.2)	–	–	4(0.9)
GII.8[P8]	–	–	–	–	–	1(1.5)	–	–	1(0.2)
GII.13[P16]	–	–	–	-	1(1.6)	-	–	–	1(0.2)
GII.17[P17]	–	–	–	1(1.6)	6(9.5)	1(1.5)	1(1.4)	3(5.1)	12(2.6)
GII.21[P21]	–	–	–	-	-	1(1.5)	–	–	1(0.2)
Total	28(100.0)	58(100.0)	57(100.0)	61(100.0)	63(100.0)	65(100.0)	72(100.0)	59(100.0)	463 (100.0)

*N* Norovirus positive numbers, *m%* Constituent ratio of each genotype

From 2012 to 2015, the constituent ratio of GII.4 Sydney[P31] increased from 22.4% in 2012 (when this genotype began to emerge) to a peak of 76.2% in 2015. Meanwhile, the constituent ratio of GII.3[P12] declined from 2013 (45.6%, 28/57) to 2015 (9.5%, 6/63). In 2016 and 2017, the constituent ratio of GII.4 Sydney[P31] declined to 55.4% (36/65) and 54.2% (39/72), respectively, while that of GII.3[P12] increased to 26.2% (17/65) in 2016 and then declined to 16.7% (12/72) in 2017, and that of GII.2[P16] increased to 27.7% (20/72) in 2017. In 2018, the constituent ratios of GII.4 Sydney[P31] and GII.3[P12] increased to 62.7% (37/59) and 22.0% (13/59), respectively. However, the constituent ratio of GII.2[P16] declined sharply to 5.1% (3/59) in 2018.

### Distribution of norovirus dual types in children of different ages

As shown in Table. 3, most dual types were detected in children aged 12 to 23 months, including all 19 dual types except GI.5[P4], GII.1[P33], GII.2[P2], GII.3[P4 2006b] and GII.7[P7], followed by children aged 6 to 11 months (10 types). GII.4 Sydney[P31] was detected in all age groups of children. GII.3[P12] and GII.4 2006b[P4 2006b] were detected in all age groups except 48 to 59 months. GII.3[P12] accounted for 22.3% (29/130) of all positive samples in children aged 0 to 5 months, followed by children aged 6 to 11 months (14.6%, 37/253), 12 to 23 months (11.6%, 37/319), 24 to 35 months (8.5%, 5/59) and 36 to 47 months (8.1%, 3/37).

**Table 3:** Norovirus dual types distribution by age group among outpatient children with diarrhea in Beijing, 2011–2018

Genotype[P-type]	0–5 N(m%)	6–11 N(m%)	12–23 N(m%)	24–35 N(m%)	36–47 N(m%)	48–57 N(m%)	Total N(m%)
GI.5[P4]	–	–	–	–	1(2.7)	–	1(0.1)
GII.1[P16]	–	–	1(0.3)	–	–	–	1(0.1)
GII.1[P33]	1(0.8)	–	–	–	–	–	1(0.1)
GII.2[P2]	–	2(0.8)	2(0.6)	-	1(2.7)	1(9.1)	6(0.7)
GII.2[P12]	1(0.8)	–	–	–	–	–	1(0.1)
GII.2[P16]	–	5(2.0)	12(3.8)	5(8.5)	1(2.7)	–	23(2.8)
GII.3[P4 2006b]	1(0.8)	1(0.4)	–	–	–	–	2(0.2)
GII.3[P12]	29(22.3)	37(14.6)	37(11.6)	5(8.5)	3(8.1)	–	111(13.7)
GII.3[P21]	1(0.8)	–	1(0.3)	–	–	–	2(0.2)
GII.4 2006b[P4 2006b]	4(3.1)	12(4.7)	15(4.7)	1(1.7)	2(5.4)	–	34(4.2)
GII.4 unassigned[P4 2006b]	–	3(1.2)	3(0.9)	2(3.3)	–	–	8(0.9)
GII.4 2009[P4 2009]	1(0.8)	1(0.4)	1(0.3)	–	–	–	3(0.4)
GII.4 Sydney[P31]	22(16.9)	75(29.6)	111(34.8)	18(30.5)	9 (24.3)	4(36.4)	239(29.5)
GII.6[P7]	2(1.5)	5(2.0)	4(1.3)	1(1.7)	–	–	12(1.5)
GII.7[P7]	–	–	–	1(1.7)	3(8.1)	–	4(0.5)
GII.8[P8]	–	–	1(0.3)	–	–	–	1(0.1)
GII.13[P16]	–	–	1(0.3)	–	–	–	1(0.1)
GII.17[P17]	–	2(0.8)	7(2.2)	–	2(5.4)	1(9.1)	12(1.5)
GII.21[P21]	–	–	1(0.3)	–	–	–	1(0.1)
Untyped	68(52.3)	110(43.5)	122(38.2)	26(44.1)	15(40.5)	5(45.5)	346(42.8)
Total	130(100.0)	253(100.0)	319(100.0)	59(100.0)	37(100.0)	11(100.0)	809(100.0)

*N* Norovirus positive numbers, *m%* Constituent ratio of each genotype

### Phylogenetic analysis based on partial capsid and RdRp genes

**Fig. 2:**
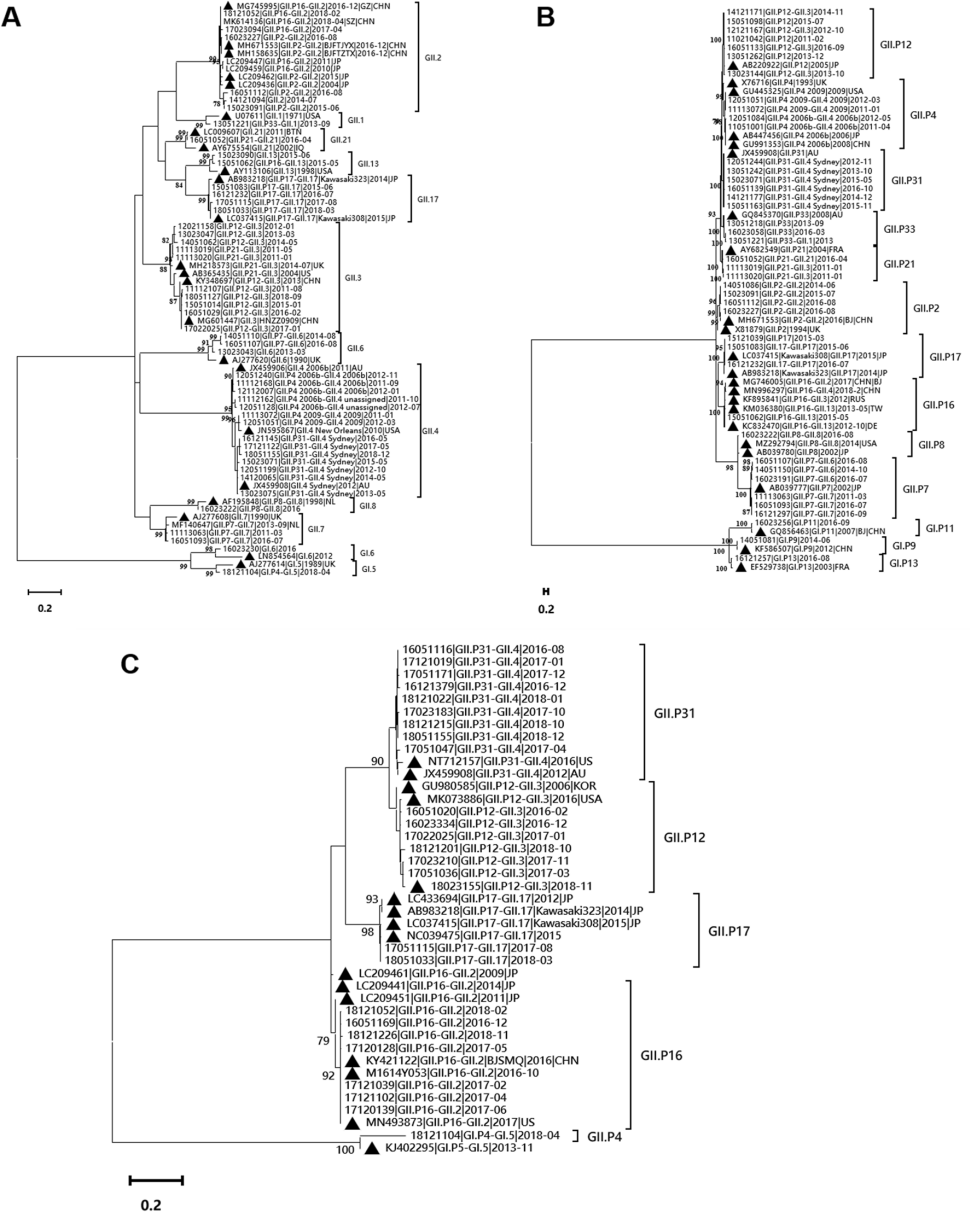
Maximum-likelihood phylogenetic analysis of norovirus based on partial capsid and RdRp genes. **A** partial capsid genes (282 bp) of norovirus strains from 2011 to 2018, **B** partial RdRp (274 bp) genes of norovirus strains from 2011 to 2015 **C** partial RdRp genes (262 bp) of norovirus strains from 2016 to 2018. The black triangles in **A**, **B** and **C** indicate reference strains. The tree was constructed in MEGA-X with 1, 000 bootstrap replicates. Bootstrap values less than 75% are not displayed. The best nucleotide substitution model which producing the lowest BIC (Bayesian information criterion) was determined by the maximum likelihood model testing tool, and the K2 + G (Kimura two-parameter method and gamma distribution) model was selected to be the best fit mode

Phylogenetic trees of representative sequences obtained in this study and reference sequences from GenBank were constructed. As shown in Fig. 2A, the 15 genotypes identified in this study were GII.1, GII.2, GII.3, GII.4 2006b, GII.4 unassigned, GII.4 2009, GII.4 Sydney, GII.6, GII.7, GII.8, GII.13, GII.17, GII.21, GI.5 and GI.6. The GII.2 strains identified in this study mainly belonged to the cluster that included the re-emerging GII.2[P16] 2016–2017, while others belonged to a clade including GII.2[P2]. However, one GII.2[P2] strain identified in this study (16023227) was distinct from other GII.2[P2] strains. This strain grouped within the same cluster as the GII.2[P2] strains MH671553 and MH158635; these three GII.2[P2] strains formed peripheral branches within the cluster including the re-emerging GII.2[P16] 2016–2017. The GII.3 strains identified in this study grouped into two clusters that included mostly strains identified in 2011–2014 and 2015–2018, respectively. Strains typed as GII.4 unassigned formed a single cluster that was most closely related to the cluster including GII.4 2006b strains. The GII.17 strains identified in this study belonged to a single cluster including the GII.17[P17] strain Kawasaki308.

A total of 14 P-types identified in this study using the primer pair 289/290 were GII.P2, GII.P4 2006b, GII.P4 2009, GII.P7, GII.P8, GII.P12, GII.P16, GII.P17, GII.P21, GII.P31, GII.P33, GI.P9, GI.P11 and GI.P13 (Fig. 2B). GII.P7 strains grouped into two clusters containing GII.6[P7] and GII.7[P7] strains, respectively. All GII.P17 strains grouped into the same cluster as the GII.17[P17] strain Kawasaki308.

Figure 2C shows a phylogenetic tree based on the 262 bp partial RdRp sequences obtained using primers Mon431/G2SKR and Mon432/G1SKR. The tree included the P-types GII.P16, GII.P12, GII.P31, GII.P17, and GI.P4. The GII.P16 strains identified from 2016 to 2018 in this study grouped into a single cluster including the re-emerging GII.2[P16] 2016–2017 strains. The GII.17[P17] strains detected in 2017 and 2018 grouped into the same cluster as the GII.17[P17] strain Kawasaki308.

## Discussion

In China, norovirus now plays an increasingly important role in the etiology of AGE [8, 22, 36]. In this study, the overall prevalence of norovirus in children younger than 5 years with diarrhea from 2011 to 2018 was 16.5% (809/4911). From January 2011 to December 2013, the prevalence of norovirus was 17.0%. Between 2014 and 2018, the overall prevalence of GI, GII and GI/GII norovirus mixed infection was 0.3%, 15.7%, and 0.1%, respectively, similar to that in Shanghai [19].

From 2011 to 2018, children aged 12–23 months had the highest norovirus prevalence, followed by children aged 6 to 11 months. This finding was consistent with some studies [5, 8, 37, 38] while some other studies reported the highest norovirus prevalence in children aged 6 to 11 months [39, 40]. Correspondingly, most dual types were detected in children aged 12–23 months, followed by children aged 6–11 months (Fig. 2). These results suggest that children aged 12–23 months are more susceptible to norovirus infection. This phenomenon in this study may be explained by two reasons. First, the immune system in children aged 12–23 months was still not perfect, and children aged 12–23 months had lower consumption of human milk which may provide protection against infection [41] than children aged 6–11 months. Second, increased exposure to noroviruses was associated with the expanded range of activities in children aged 12–23 months, and these children have poor health awareness.

No significant differences were found between the positive rates in different years in this study (Table 1). The positive rate in 2011 was relatively higher than those in other years. The positive rate in 2012 was 17.2%, then the positive rates in 2013–2016 decreased to 14.9%–15.6%. These results have two explanations. First, as shown by Gao [28], between September 2012 and March 2013, GII.4 Sydney variant emerged as the predominated genotype and increased norovirus activity. Second, GII.4 Sydney specific herd immunity in children lasted during the next seasons [16], thereby influencing the positive rates. Although the GII.17[P17] 2014–2015 strains emerged as the predominant genotype among AGE outbreaks in Beijing between December 2014 and March 2015 [23], this genotype didn’t influence the positive rates of norovirus infection in sporadic children aged < 5 years obviously in 2014–2015, and GII.17[P17] accounted for 1.6% and 9.5% of genotyped strains in 2014 and 2015, respectively. Other studies reported that children aged < 5 years accounted for 15.6% and 0.0% of GII.17[P17] cases in Hong Kong [42] and Shanghai [43], respectively, during 2014–2015.

In general, most norovirus cases occurred in cooler months (October to March in the Northern Hemisphere) with a peak in winter months (December to February in the Northern Hemisphere) [44]. The seasonality of norovirus diarrhea in this study agreed with the general pattern of most cases occurring in cooler months. However, the peak prevalence occurred in autumn, followed by winter, spring, and summer. This finding was similar to that reported in Shanghai [19].

GII.4 is the only norovirus genotype associated with global pandemics [45]. Since 1995, GII.4 noroviruses have been responsible for > 80% of all human norovirus infections worldwide [45]. Six GII.4 variants have been associated with global pandemics: US96, Farmington Hills 2002, Hunter 2004, Den Haag 2006b, New Orleans 2009, and, most recently, Sydney 2012 [45]. The evolution of GII.4 noroviruses is driven by antigenic drift and recombination [46]. In China, the GII.4 2006b strains and GII.4 2009 strains, which predominated prior to 2012, were replaced by GII.4 Sydney strains [19, 47]. From 2013 to 2018, GII.4 Sydney[P31] was the predominant dual type.

On the one hand, although GII.4 Sydney[P31] was the most common norovirus in sporadic pediatric cases in this study in Beijing and many other countries [4, 19], studies of norovirus outbreaks in China from 2016 to 2018 [18] and norovirus outbreaks in Beijing from 2014 to 2017 [23] suggested that the GII.4 Sydney[P31] norovirus accounted for very small proportions of norovirus outbreaks in kindergartens. On the other hand, GII.2[P16] 2016–2017 strains and GII.17[P17] 2014–2015 strains, the most predominant causes of norovirus outbreaks in the seasons of 2016–2017 and 2014–2015 respectively [23], ranked as the second most prevalent genotypes in 2017 and 2015 in this study. Several explanations have been considered for this phenomenon. First, GII.4 noroviruses can bind to a wider range of histo-blood group antigens (HBGAs) and thus have a larger population susceptible to infection [48]. Second, children with GII.4 noroviruses were reported to have more severe illness including prolonged diarrhea and vomiting [17], potentially resulting in more outpatients. Third, GII.4 noroviruses have been circulating in human populations for many years, the high prevalence of GII.4 noroviruses in sporadic cases may have led to enhanced genotype specific herd immunity [16] which may lead to their low prevalence in norovirus outbreaks [18, 23]. Fourth, the sporadic cases cover people under 5 years of age, while the outbreaks in kindergarten and primary school children are mostly older than sporadic cases. Newborns and younger children who have not been infected with GII.4 lack of corresponding immune protection. Moreover, in norovirus outbreaks [23], most cases of GII.2[P16] 2016–2017 and GII.17[P17] 2014–2015 had vomiting but no diarrhea and they didn’t go to the hospital; even if they went to the hospital, fecal samples would not be collected. Thus, GII.2[P16] 2016–2017 and GII.17[P17] 2014–2015 were less detected in sporadic cases of pediatrics.

GII.3[P12] was the second most predominant norovirus genotype detected in this study, and it was the only genotype detected in each year from 2011 to 2018, indicating that GII.3 noroviruses are common causes of sporadic pediatric infection [17, 49]. As reported by Jin, during 2016–2018, GII.3[P12] caused higher proportional number of norovirus outbreaks in kindergarten (6.8%) than GII.4 Sydney[P31] (1.4%) [18], reflecting the relatively lower level of herd immunity against GII.3[P12] in children compared with GII.4 Sydney[P31]. Phylogenetic analysis of partial VP1 nucleotide sequences of GII.3[P12] revealed that this genotype formed a distinct group in Beijing from 2015 to 2018. Genetic changes in GII.3[P12] strains may have contributed to the prevalence of this genotype in both sporadic cases and outbreaks among children < 5 years of age in Beijing.

During the 2014–2015 season, the GII.17[P17] strains emerged as the predominant cause of AGE outbreaks and sporadic cases in parts of Asia [50, 51]. Unlike GII.4, the GII.17 strain was rarely detected in human cases between its first description in 1978 and 2014. Previous evolutionary analyses showed that from 2013 to 2017, the GII.17[P17] strains formed two clusters (the Kawasaki308 type and the Kawasaki323 type). The Kawasaki308 type dominated from 2014 to 2016 [50]. In this study, all eight GII.17 strains were identified as the Kawasaki308 type. Structural analysis of GII.17 noroviruses showed that the increased prevalence of the GII.17 2014–2015 strains might be related to the V444Y mutation, which optimized the HBGA binding site and enhanced HBGA binding [52].

The prevalence of GII.17[P17] 2014–2015 decreased notably later. During the winter season of 2016–2017, the GII.2[P16] strains reemerged as the predominant cause of norovirus outbreaks in China and other countries [23, 25, 53–55]. In this study, the GII.2[P16] strains were only detected in 2017 and 2018. The other genotype combination of GII.2 norovirus detected in this study was GII.2[P2] and was only detected occasionally from 2014 to 2016. As shown in Fig. 2A, the partial VP1 gene sequence of GII.2[P2] strain 16023227 detected in the Xicheng district of Beijing was distinct from those of other GII.2[P2] strains and was most closely related to the emerging GII.2[P2] strain BJFTZTX (accession number MH671553) which was identified from a norovirus outbreak in Fengtai district in December 2016 [56]. The full-length capsid gene of 16023227 was also obtained and it was confirmed to be grouped together with the emerging GII.2[P2] strains like BJFTZTX (data not shown). As shown by Ao [56], the emerging GII.2[P2] strains and the re-emerging GII.2[P16] strains have identical VP1 gene sequences. However, in 2016 and 2017, the re-emerging GII.2[P16] strains became the predominant genotype, while the emerging GII.2[P2] strains did not increase in prevalence. This finding may imply that the GII.P16 polymerase may positively influence viral fitness without modification of the capsid proteins [57].

It is a limiting factor in this study that the number of cases used for genotyping and phylogenetic analysis in this study was about 60% of the total number of positive cases, thus the prevalence of genotypes may not be fully reflected.

## Conclusions

This study analyzed the epidemiological characteristics and genetic diversity of noroviruses from outpatient children < 5 years old in Beijing from 2011 to 2018. The genotype diversity of noroviruses among outpatient children < 5 years of age differed from that of noroviruses causing outbreaks in Beijing. Further detailed comparisons of genetic diversity of noroviruses between sporadic norovirus cases and outbreaks, and those between sporadic cases of children (< 5 years old), adolescents (≥ 5 years old, < 18 years old) and adults (≥ 18 years old) are necessary. Complete genome sequencing of norovirus genotypes is needed to better understand the evolutionary mechanisms of noroviruses and their relationships with epidemiologic features.

## Data Availability

All data involved in this study is available upon reasonable request made to the corresponding author.

## Abbreviations

AGE: Acute gastroenteritis
ORF: Open reading frame
RdRp: RNA-dependent RNA polymerase
RT-PCR: Reverse transcription polymerase chain reaction
VP1: Major capsid protein
CDC: Centers for Disease Control and Prevention
HBGA: Histo-blood group antigen
